# Malignant Catatonia From Paliperidone Withdrawal With Successful Pharmacological Treatment: A Diagnostic and Therapeutic Challenge

**DOI:** 10.7759/cureus.108510

**Published:** 2026-05-08

**Authors:** Muhammad Usman, Muhammad Usman Shahbaz, Laiba Murtaza, Muhammad Zawar Asif, Mohankumar Doraiswamy

**Affiliations:** 1 Internal Medicine, Mercy Hospital Fort Smith, Fort Smith, USA; 2 Internal Medicine, Arkansas College of Osteopathic Medicine, Fort Smith, USA; 3 Medicine, King Edward Medical University, Lahore, PAK; 4 Internal Medicine and Critical Care, Mercy Hospital Fort Smith, Fort Smith, USA

**Keywords:** benzodiazepine use in catatonia, electroconvulsive therapy (ect), long-acting injectable antipsychotics, malignant catatonia, paliperidone side effect, patient nonadherence, schizophrenia and other psychotic disorders

## Abstract

Catatonia is a neuropsychiatric syndrome of psychomotor disturbances occurring in various psychiatric and medical conditions, as well as from adverse effects of antipsychotics. Malignant catatonia is a severe, life-threatening subtype characterized by autonomic instability along with typical catatonia features. It requires immediate treatment with electroconvulsive therapy (ECT) in critical care settings. While oral antipsychotics are well-recognized causes of withdrawal catatonia, cases related to long-acting injectable antipsychotic drugs causing withdrawal catatonia occur less commonly.

We present a case of a 43-year-old man with schizophrenia, major depressive disorder, and generalized anxiety disorder who had been on paliperidone. The patient presented to the hospital after missing a dose of paliperidone with symptoms consistent with catatonia. His condition rapidly deteriorated, leading to malignant catatonia. The ECT plan was deferred due to non-availability at the facility and the family’s wishes. The patient was treated with high-dose benzodiazepines, which successfully improved his symptoms. He had a prolonged hospital stay due to slow recovery and was discharged on a long taper of benzodiazepines. Paliperidone was resumed as an outpatient because of concerns about rebound catatonia following immediate resumption during hospitalization.

This case highlights several atypical features related to catatonia and long-acting injectable antipsychotic medications. It also emphasizes the importance of potential adverse effects from medication nonadherence, especially with long-acting injectable antipsychotics, which can pose diagnostic challenges. Early recognition of malignant hyperthermia, aggressive benzodiazepine dose escalation, and ECT remain cornerstones of successful management. The case further highlights therapeutic challenges in psychiatric emergencies due to patient and system factors.

## Introduction

Catatonia is a neuropsychiatric syndrome of psychomotor disturbances. The Diagnostic and Statistical Manual of Mental Disorders, 5th Edition (DSM-5) defines catatonia as the presence of at least 3 of 12 psychomotor features: stupor, cataplexy, waxy flexibility, mutism, negativism, mannerisms, stereotypies, agitation, grimacing, echolalia, and echopraxia [1]. It can occur with various psychiatric disorders, such as schizophrenia, mood disorders, electrolyte imbalances, and antipsychotic drug toxicity or withdrawal [1,2].

Malignant catatonia is a severe, life-threatening form of catatonia characterized by autonomic instability in addition to features of psychomotor disturbance. It has a mortality rate of 50% or more if left untreated [3].

While antipsychotic drugs remain the primary treatment for schizophrenia and associated disorders, their discontinuation is a well-recognized cause of relapse of psychiatric symptoms, with nonadherence being the most common cause. To address this challenge, long-acting injectable antipsychotics such as paliperidone were developed, eliminating the need for daily dosing [1].

Although catatonia has been well-reported following withdrawal from oral antipsychotic drugs such as clozapine [4], the phenomenon of catatonia resulting from nonadherence to long-acting antipsychotics remains poorly characterized in the literature. Furthermore, the development of malignant catatonia after withdrawal of long-acting injectable antipsychotic medications is the least reported, which adds to diagnostic and therapeutic challenges.

Electroconvulsive therapy (ECT) is the definitive and most effective treatment for malignant catatonia. Recently, pharmacological treatments have shown successful symptom resolution in some case studies [3]. Recently, drugs such as memantine, amantadine, valproic acid, carbamazepine, and bromocriptine have been used in nonmalignant catatonia when lorazepam or ECT fails or is unavailable. A limited review of pharmacological outcomes in malignant catatonia highlights therapeutic challenges and resource constraints, especially when ECT is unavailable or when family preferences are involved [3].

Here, we present a case of a patient with a history of schizophrenia being treated with monthly infusions of long-acting injectable antipsychotic medication, paliperidone. The patient developed malignant catatonia following nonadherence to his medication regimen, highlighting the importance of medication review, recognizing potential complications from paliperidone withdrawal, and the use of aggressive benzodiazepine therapy for malignant catatonia where ECT is not available.

## Case presentation

A 43-year-old man with schizophrenia, major depressive disorder, generalized anxiety disorder, and hypertension was brought to the hospital by family after several days of decreased functional status and responsiveness over the past several days. Family reported poor oral intake, reduced interaction, and medication nonadherence to his antihypertensives and oral as well as injectable antipsychotics as a prominent part of his medical history. No prior history of such a clinical presentation were reported.

The patient also had a recent hospitalization about two weeks ago for community-acquired pneumonia, which improved with empiric antibiotic treatment, and he was discharged in stable condition at that time and eventually returned to his baseline mentation.

At the time of presentation, the patient was awake but minimally responsive to verbal commands. Vital signs were stable except for persistent tachycardia. On physical examination, the patient was noted to have lethargic, minimal response to verbal commands by just nodding his head, flat affect, mumbled speech, waxy flexibility, and psychomotor retardation, along with tachycardia. No muscle rigidity or hyperreflexia was noted.

Initial laboratory work, including cell count, metabolic profile, thyroid-stimulating hormone, serum ammonia, vitamin B12, folic acid, serum ethanol, and creatinine phosphokinase (CK) levels, was unremarkable. A urine drug screen was also negative. Infectious evaluation, including respiratory pathogen polymerase chain reaction (PCR) testing and blood cultures, was also negative. Cerebrospinal fluid (CSF) analysis after lumbar puncture was done, and CSF culture and Gram stain were also negative (Table 1).

**Table 1:** Laboratory evaluations WBC: white blood cell, RBC: red blood cell, Hgb: hemoglobin, Hct: hematocrit, MCV: mean corpuscular volume, MCH: mean corpuscular hemoglobin, MCHC: mean corpuscular hemoglobin concentration, RDW: red cell distribution width, Plt: platelets, ANC: absolute neutrophil count, ALC: absolute lymphocyte count, BUN: blood urea nitrogen, AST: aspartate aminotransferase, ALT: alanine aminotransferase, ALP: alkaline phosphatase, eGFR: estimated glomerular filtration rate, PCR: polymerase chain reaction, TSH: thyroid-stimulating hormone, CK: creatine kinase, CSF: cerebrospinal fluid

Laboratory test	Result	Reference range
Complete blood count/metabolic panel		
WBC	8.8 K/µL	4.2-9.1 K/µL
RBC	4.07 M/µL	4.63-6.08 M/µL
Hemoglobin	11.8 g/dL	13.7-17.5 g/dL
Hematocrit	34.4%	40.1%-51.0%
MCV	84.5 fL	79.0-92.2 fL
MCH	29.0 pg	25.7-32.2 pg
MCHC	34.3 g/dL	32.3-36.5 g/dL
RDW	14.8%	11.6%-14.4%
RDW-SD	45.1 fL	35.1-43.9 fL
Platelets	276 K/µL	163-337 K/µL
Neutrophils	68%	34%-71%
Lymphocytes	22%	19%-53%
Monocytes	9%	5%-13%
Eosinophils	0%	1%-7%
Basophils	1%	0%-1%
Immature granulocytes	0%	0%-1%
Neutrophil, absolute	6.02 K/µL	1.05-6.10 K/µL
Lymphocyte, absolute	1.91 K/µL	1.10-3.70 K/µL
Monocyte, absolute	0.78 K/µL	0.20-0.80 K/µL
Eosinophil, absolute	0.02 K/µL	0.00-0.50 K/µL
Basophil, absolute	0.04 K/µL	0.00-0.08 K/µL
Immature granulocyte, absolute	0.03 K/µL	0.00-0.31 K/µL
Sodium	138 mmol/L	136-145 mmol/L
Potassium	4.3 mmol/L	3.5-5.1 mmol/L
Chloride	101 mmol/L	98-107 mmol/L
CO₂	25 mmol/L	22-29 mmol/L
Calcium	10.2 mg/dL	8.6-10.0 mg/dL
BUN	12 mg/dL	6-20 mg/dL
Creatinine	0.67 mg/dL	0.67-1.17 mg/dL
Glucose	97 mg/dL	74-99 mg/dL
Total protein	7.1 g/dL	6.6-8.7 g/dL
Albumin	3.9 g/dL	3.5-5.2 g/dL
Total bilirubin	0.5 mg/dL	0.0-1.2 mg/dL
Alkaline phosphatase	63 U/L	40-129 U/L
AST	22 U/L	0-50 U/L
ALT	10 U/L	0-50 U/L
eGFR	>60 mL/min/1.73 m²	≥60 mL/min/1.73 m²
Additional tests		
Respiratory pathogen PCR	Not detected	Not detected
COVID-19 PCR	Not detected	Not detected
Vitamin B12	740 pg/mL	232-1,245 pg/mL
Folate, serum	13.1 ng/mL	>4.5 ng/mL
TSH	2.14 µIU/mL	0.27-4.20 µIU/mL
CK	254 U/L	39-308 U/L
CSF analysis		
CSF appearance	Clear	Clear
CSF color	Colorless	Colorless
CSF WBC	<3/µL	0-5/µL
CSF RBC	<2,000/µL	0-5/µL
Protein, CSF	37.4 mg/dL	15-45 mg/dL

**Figure 1:**
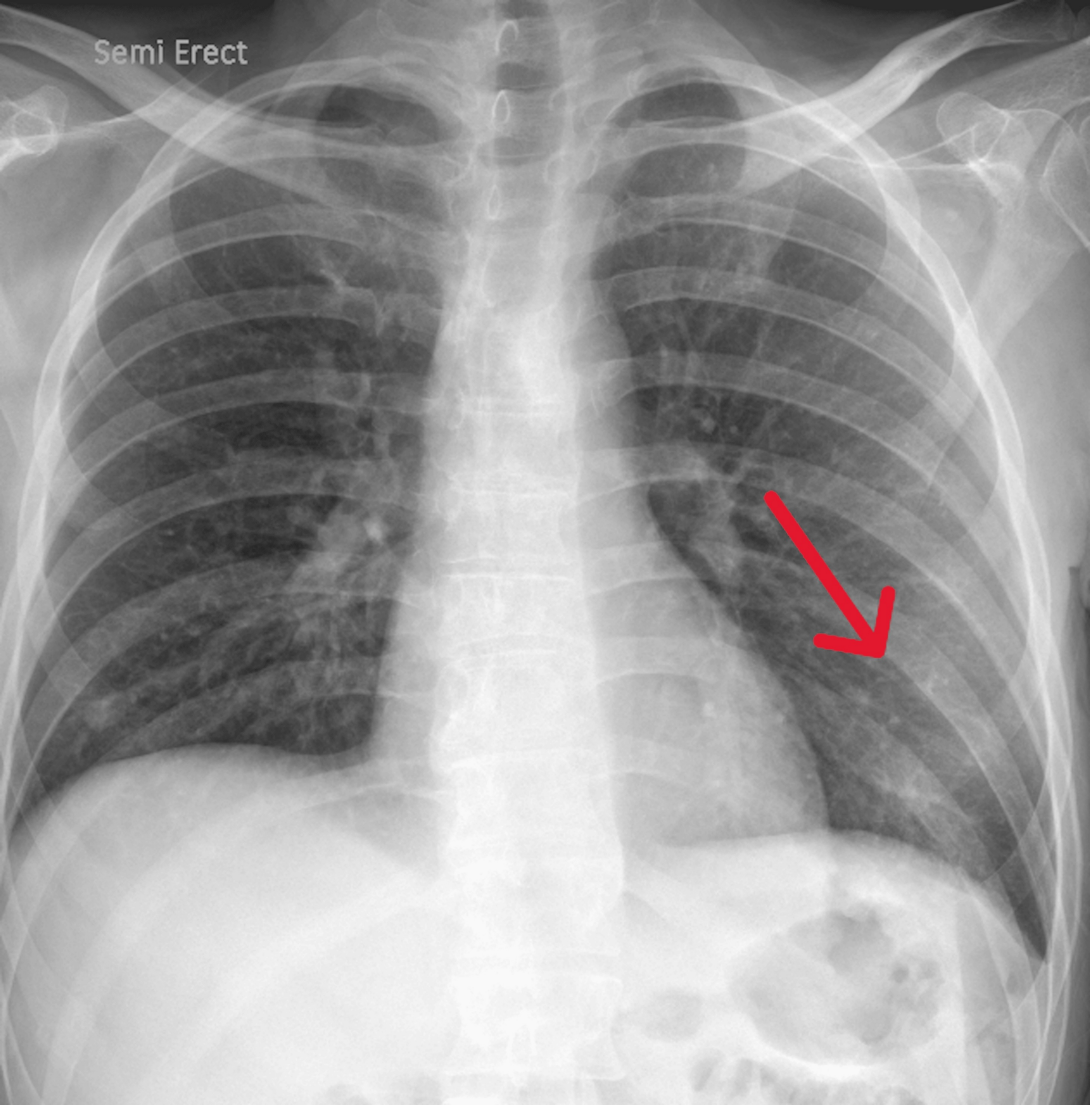
X-ray of the chest showing left lobe infiltrates (red arrow) from previous infection

Chest X-ray revealed lung infiltrates from a previous lung infection (Figure 1).

**Figure 2:**
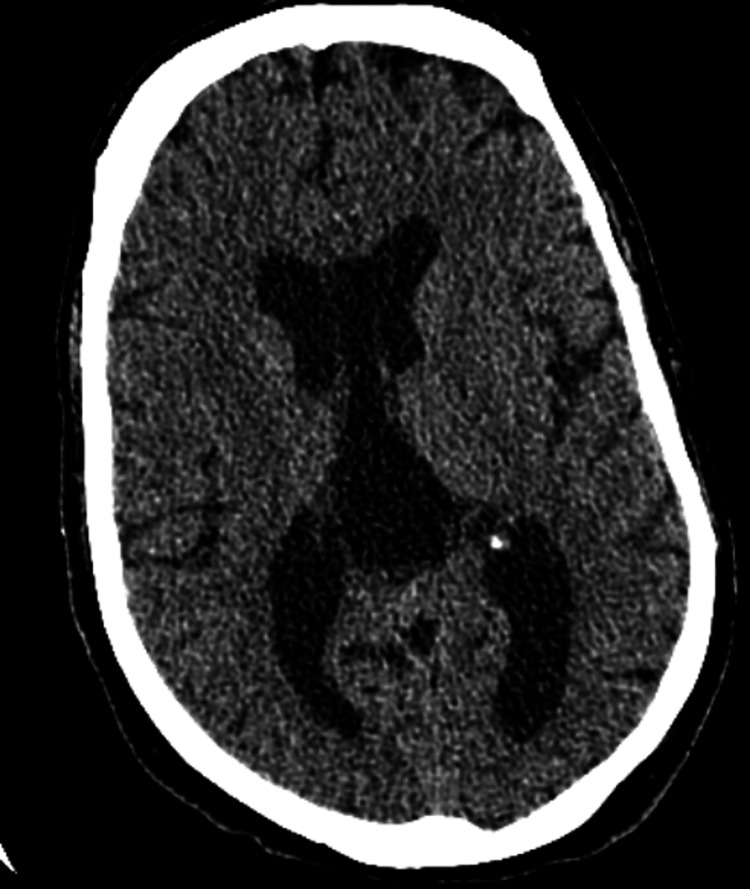
CT scan of the head without contrast negative for any infarct, hemorrhage, or mass effect

**Figure 3:**
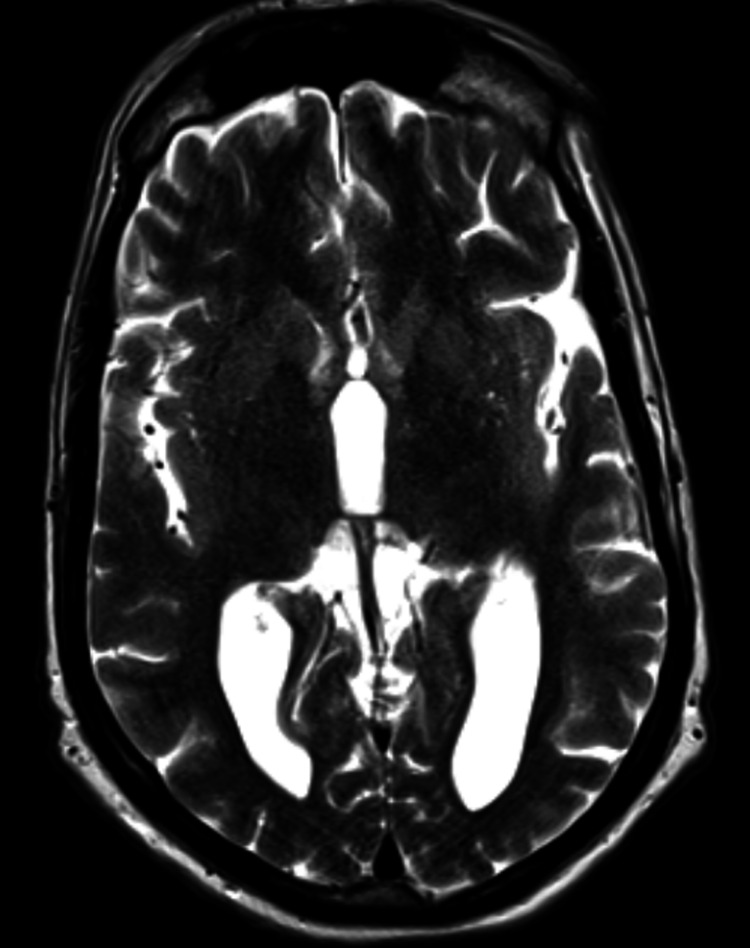
MRI of the brain without contrast (T2 sequence) negative for any mass, infarct, or demyelinating lesions

CT scan of the head without contrast (Figure 2) and a follow-up MRI of the brain without contrast (Figure 3) were unremarkable for any infarct, hemorrhage, or intracranial mass.

An electroencephalogram showed mild generalized background slowing without any focal or epileptiform changes.

Given a negative infectious, metabolic, and neurological workup, the patient was evaluated by the inpatient psychiatric team, and he met DSM-5 criteria for catatonia (Bush-Francis Catatonia Rating Scale (BFCRS) [5] score: 17) (Table 2).

**Table 2:** BFCRS BFCRS: Bush-Francis Catatonia Rating Scale Source: [5]

BFCRS Item	Score (points)
Excitement	0
Immobility/stupor	2
Mutism	3
Staring	2
Posturing/catalepsy	0
Grimacing	1
Echopraxia/echolalia	0
Stereotypy	0
Mannerisms	0
Verbigeration	0
Rigidity	1
Negativism	0
Waxy flexibility	3
Withdrawal	3
Impulsivity	0
Automatic obedience	0
Gegenhalten	0
Grasp reflex	0
Perseveration	0
Combativeness	0
Autonomic abnormality	2
Total BFCRS score	17

A diagnostic breakthrough occurred when a family at bedside reported that the patient had missed his last monthly infusion of paliperidone scheduled around three weeks ago due to his history of nonadherence. At that time, the patient was challenged with IV lorazepam 2 mg with slight improvement initially; however, the patient deteriorated quite rapidly after that with worsening mentation and had to be admitted to the ICU for airway protection and possible need for intubation. The patient also developed tachycardia with a heart rate of 120-145 beats per minute and a low-grade fever of 100.8°F. Due to concerns of developing malignant catatonia due to new-onset autonomic instability, the patient was challenged with a higher dose of IV lorazepam 4 mg per psychiatry recommendations. ECT was also considered if the lorazepam challenge failed, per the patient’s family’s preferences, due to the non-availability of ECT at the current facility. The patient’s mentation and autonomic instability drastically improved on a higher dose of IV lorazepam, further strengthening the suspicion of malignant catatonia. Due to the patient’s clinical improvement, the decision for electroconvulsive therapy was subsequently deferred. The patient was eventually started on scheduled IV lorazepam 4 mg every 6 hours, totaling up to 16 mg/day in the ICU. His encephalopathy and psychomotor retardation gradually improved over the course of the next two weeks. A shared decision was made to discharge the patient on maintenance therapy of lorazepam 2 mg every 8 hours over the course of the next 3 months, with a plan to resume paliperidone on an outpatient basis with close follow-up with psychiatry. The patient continued his maintenance therapy of lorazepam with resumption of paliperidone on an outpatient basis. At the last follow-up, his symptoms of schizophrenia were stable with no recurrence of catatonic symptoms.

## Discussion

This case highlights several important clinical aspects related to malignant catatonia, long-acting injectable antipsychotics, the requirement of dose escalation of benzodiazepines in severe catatonia, and the use of pharmacological treatment in psychiatric emergencies such as malignant catatonia in settings where ECT is not available and not desired by the family. The development of autonomic instability, in addition to characteristic features of catatonia, transformed his case from a challenging psychiatric syndrome into a psychiatric emergency, leading to further diagnostic and therapeutic challenges. The patient had progression from simply catatonia to malignant catatonia involving autonomic instability, which highlights the importance of vigilant monitoring for autonomic dysfunction in all patients presenting with catatonia.

Although there are no standard criteria for malignant catatonia, it has traditionally been defined as a state of autonomic instability in addition to the classic features of catatonia [6].

The patient’s rapid deterioration after a transient improvement of symptoms from a low-dose lorazepam challenge, subsequently leading to the development of malignant catatonia, represents an important turning point in our case. It emphasizes the need for dose escalation of benzodiazepines in clinical situations where catatonia is rapidly transforming into a malignant subtype [1,6].

Malignant catatonia must always be distinguished from neuroleptic malignant syndrome (NMS) as this carries important therapeutic implications. Neuroleptic malignant syndrome typically presents with rigidity, hyperthermia, mutism, delirium, and autonomic instability [1].

A systematic review examining the clinical overlap between antipsychotic-related catatonia and NMS found substantial overlap with at least 11 variables demonstrating status to goal significance. Among those variables, diaphoresis, rigor, fever, tremors, laboratory evidence of muscle injury, and leukocytosis favored NMS, while negativism, waxy flexibility, stupor, and stereotypy were indicative of catatonia [7]. In this case, withdrawal of antipsychotic medication due to nonadherence, psychiatric history, absence of muscle rigidity, normal reflexes and creatine kinase levels, and lack of recent antipsychotic exposure collectively support a diagnosis of catatonia over NMS.

The association between antipsychotic withdrawal and the development of catatonia remains under-reported in the literature compared to antipsychotic-induced catatonia. Recently, there has been increasing literature regarding oral antipsychotic withdrawals related to catatonia. A case report of clozapine withdrawal-induced catatonia was described in a patient who developed symptoms consistent with catatonia a week after cessation of clozapine. The patient’s symptoms of catatonia improved significantly after reintroduction of clozapine in the hospital [4].

Such cases related to withdrawal catatonia from long-acting injectable antipsychotic medications remain even rarer in the literature, which adds further to the diagnostic and therapeutic dilemma of this case.

Although the ECT remains the first line for treatment of malignant catatonia [1], it becomes challenging in psychiatric emergencies such as malignant catatonia at places where such facilities are not available, and family preferences are also against transfer to another facility for the ECT. Based on limited literature, the patient was treated with high-dose benzodiazepines, which have been successful in a few cases reported in the literature with similar patient and system factors [3].

In continuation of therapeutic considerations, typically, maximal therapeutic response occurs within 3-7 days of initiating benzodiazepines; it may take longer in some patients, as evidenced in our case, where expected response patterns occurred gradually over two weeks [8].

Paliperidone was not resumed during hospitalization due to the previously reported phenomenon of rebound catatonia after resuming paliperidone in patients who initially improved from catatonia; however, symptoms recurred after resuming paliperidone during the same hospitalization [9].

Management of malignant catatonia extends beyond pharmacological intervention and should include close monitoring of vital signs, ECT whenever possible, and close airway monitoring, given its high mortality [6].

Discharge plan includes lorazepam maintenance therapy and gradual taper consistent with current recommendations for preventing catatonia recurrence and highlights a conservative approach well-suited for patients who developed malignant catatonia [7].

Recent evidence also suggests that lorazepam responders may have higher relapse risk [1,7]. While long-acting antipsychotic medications remain an appropriate option for patients with schizophrenia and a history of nonadherence, their use during the acute treatment of malignant catatonia remains an area of concern [1,9].

Considering the abovementioned facts, the shared decision to resume paliperidone on an outpatient basis underscores the importance of incorporating patient preferences alongside available evidence in medical decision-making. The successful resumption of paliperidone in our case without catatonia recurrence may suggest a protective role for antipsychotics. However, this remains a poorly understood phenomenon and warrants further research.

## Conclusions

This case highlights several key clinical implications. First, clinicians should maintain a high index of suspicion for catatonia, including malignant catatonia, in patients with a history of medication nonadherence and psychiatric illness undergoing antipsychotic therapy. It underscores the necessity of a thorough medical assessment and medication review at initial hospital admission. It further demonstrates the importance of monitoring lorazepam dosage increases and being alert to signs of autonomic instability in rapidly progressing catatonia. Although ECT is the definitive intervention for malignant catatonia, reports of successful pharmacological management are sparse due to patient and system variables; this remains a topic for future investigation. Finally, a gradual and extended lorazepam taper, with reintroduction of paliperidone after catatonia resolution in outpatient settings, offers an effective strategy for preventing recurrence while treating underlying psychiatric disorders.
